# Quantitative assessment of the association between *GRIA1* polymorphisms and migraine risk

**DOI:** 10.1042/BSR20181347

**Published:** 2018-12-14

**Authors:** Xueren Gao, Jianguo Wang

**Affiliations:** Department of Pediatric Endocrinology/Genetics, Shanghai Institute for Pediatric Research, Xinhua Hospital, School of Medicine, Shanghai Jiao Tong University, Shanghai 200092, China

**Keywords:** GRIA1, migraine, polymorphism, risk

## Abstract

Purpose: The association between *GRIA1* rs548294 G>A and rs2195450 C>T polymorphisms and migraine risk has been reported in several case–control studies. However, the results of studies are inconsistent. Thus, we conducted a meta-analysis to more precisely estimate the association of the two polymorphisms with migraine risk.

Methods: Eligible studies were retrieved and screened from the online databases (EMBASE, PubMed, Web of Science, Wanfang, and Chinese National Knowledge Infrastructure). The pooled odds ratio (OR) with corresponding 95.0% confidence intervals (CIs) was assessed using random- or fixed-effects model.

Results: A total of 1233 cases and 1374 controls from four eligible studies were included. The pooled analysis showed that *GRIA1* rs548294 G>A polymorphism was not significantly associated with migraine risk. *GRIA1* rs2195450 C>T polymorphism was significantly associated with migraine risk under heterozygous model (CT vs. CC, OR = 1.23, 95%CI = 1.02–1.48, *P*_Z_ = 0.03). Further subgroup analysis based on ethnicity showed a significant association of *GRIA1* rs2195450 C>T polymorphism with migraine risk in Asian population, but not in Caucasian population.

Conclusions: Our results indicates that *GRIA1* rs2195450 C>T polymorphism is significantly associated with migraine risk. However, the number of studies included in the meta-analysis was small. Thus, more high quality case–control studies with a large sample size are still required to confirm these findings.

## Introduction

Migraine is a common neurovascular disorder affecting over 17% of women and 5–8% of men around the world [1]. This disorder is characterized by recurrent moderate-to-severe headaches, and often accompanied by nausea, vomiting, photo- and phonophobia [2]. Clinically, migraine is divided into two main subtypes: migraine with aura (MA) and without aura (MO). MA is an idiopathic, recurrent disorder related to neurologic symptoms that are localized to the cerebral cortex or brain stem. MO is an idiopathic, recurring headache disorder typified by painful attacks lasting 4–72 h [3]. Although the pathogenesis of migraine is still unclear, studies suggests that inheritance factor plays an important role in the occurrence of this disease [4–6].

*GRIA1* gene is located on chromosome 5q33.2 and belongs to a family of α-amino-3-hydroxy-5-methyl-4-isoxazole propionate (AMPA) receptors. This gene encodes a subunit of ionotropic glutamate receptor regulating neuronal excitability. Synaptic hyperexcitability has been recognized as one major factor causing migraine [7]. Glutamate is an excitatory neurotransmitter in the mammalian central nervous system and can activate the trigeminovascular system and increase the risk of cortical spreading depression, which plays an important role in the pathophysiology of migraine [8–10]. Therefore, *GRIA1* gene was inferred as a migraine-related gene. Single-nucleotide polymorphism (SNP) is one of the most common forms of human DNA variations. The potentially functional SNPs in migraine-related genes have been reported to be associated with migraine risk [4–6]. Thus *GRIA1* polymorphisms may also be involved in migraine risk. In order to confirm this hypothesis, some case–control studies were conducted to investigate the association of *GRIA1* polymorphisms (rs548294 G>A, rs2195450 C>T) with migraine risk. However, the results were inconsistent [7,11–13]. To get a more accurate result, we collected all eligible studies and conducted the present meta-analysis.

## Materials and methods

### Search strategy and selection criteria

The online databases including EMBASE, PubMed, Web of Science, Wanfang, as well as Chinese National Knowledge Infrastructure (CNKI) were searched in order to obtain all the relevant articles up to August 3, 2018. Keywords for search were: ‘glutamate ionotropic receptor AMPA type subunit 1 or GRIA1’, ‘polymorphism or SNP or variation’, and ‘migraine or headache’. In addition, reference lists in retrieved articles were also screened. All included studies were conducted on human subjects, and published in either English or Chinese.

All studies selected for further meta-analysis should meet the following criteria: (i) case–control studies investigating the association between *GRIA1* polymorphisms (rs548294 G>A, rs2195450 C>T) and migraine risk; (ii) available data of genotype and allele distributions in cases and controls.

### Data extraction and quality assessment

According to the selection criteria, two authors (Gao, X.R. and Wang, J.G.) carefully reviewed all included articles and independently extracted the relevant information, including the first author’s name, year of publication, country of origin, genotyping method, the total number of case and control, and the genotype and allele frequency distribution in case and control, and *P* value of HWE in the controls. In addition, quality assessment of each included study was conducted using the Newcastle–Ottawa Scale (NOS), which included three assessment categories (selection, comparability, and exposure) [14]. The categories for selection, comparability and exposure contained four, one and three items, respectively. In the selection and exposure categories, each eligible item was endowed with a star. In the comparability category, eligible item was endowed with at most two stars. Finally, any disagreements between two authors were solved by discussion.

### Statistical analysis

For each study, the odds ratio (OR) and corresponding 95% confidence intervals (CIs) were calculated to assess the migraine risk associated with *GRIA1* rs548294 G>A and rs2195450 C>T polymorphisms. The pooled ORs were calculated under the following genetic models: heterozygous model, recessive model, homozygous model, dominant model, and allelic contrast model**.** The significance of the pooled OR was examined by the *Z*-test, and *P*_Z_ <  0.05 was considered statistically significant. Heterogeneity between studies was estimated by chi-square-based *Q*-test, and *P*_H_ < 0.1 was considered statistically significant. The random-effects model was adopted to analyze the pooled ORs if *P*_H_ value was less than 0.1. Otherwise, a fixed-effects model was exerted. Sensitivity analysis was used to assess the consistence and stability of the present meta-analysis. Additionally, funnel plots were used to estimate publication bias. All the statistical analyses were implemented with the Review Manager software (Version 5.2; Cochrane Collaboration, London, U.K.).

## Results

### Characteristics of included studies

**Figure 1:**
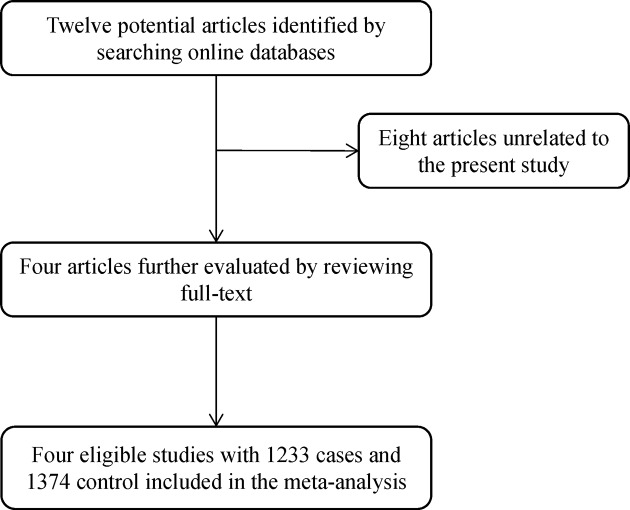
Flow chart of literature search

After an initial search, 12 potential articles were identified for further analysis. Eight articles unrelated to the present study were excluded after reviewing titles and abstracts. Finally, four eligible articles were included in the meta-analysis (Figure 1). Tables 1 and 2 presented the characteristics and quality of the included studies, respectively. Of the 4 studies with 1233 cases and 1374 controls, 1 was from Asian population, 3 were from Caucasian population.

**Table 1:** The main characteristics of the included studies in the meta-analysis

First author	Year	Country	Ethnicity	Genotyping method	Cases	Controls
Jie Fang	2016	China	Asian	Multiplex SNaPshot	331	330
Sarah Cargnin	2014	Italy	Caucasian	TaqMan	186	312
Bridget H. Maher	2013	Australia	Caucasian	PCR-RFLP	472	472
Daniela Formicola	2010	Italy	Caucasian	PCR-RFLP	244	260

Abbreviation: PCR-RFLP, polymerase chain reaction-restriction fragment length polymorphism.

**Table 2:** The quality assessment of all included studies based on the Newcastle–Ottawa Scale

Categories	Items	Fang’s study	Cargnin’s study	Maher’s study	Formicola’s study
Selection	Adequacy of case definition	*	*	*	*
	Representativeness of the cases	-	-	*	*
	Selection of controls	*	*	*	*
	Definition of controls	*	*	*	*
Comparability	Comparability of cases/controls	*	*	*	*
Exposure	Ascertainment of exposure	*	*	*	*
	Same method of ascertainment for cases and controls	*	*	*	*
	Non-response rate	*	*	*	*

### Meta-analysis results

**Figure 2:**
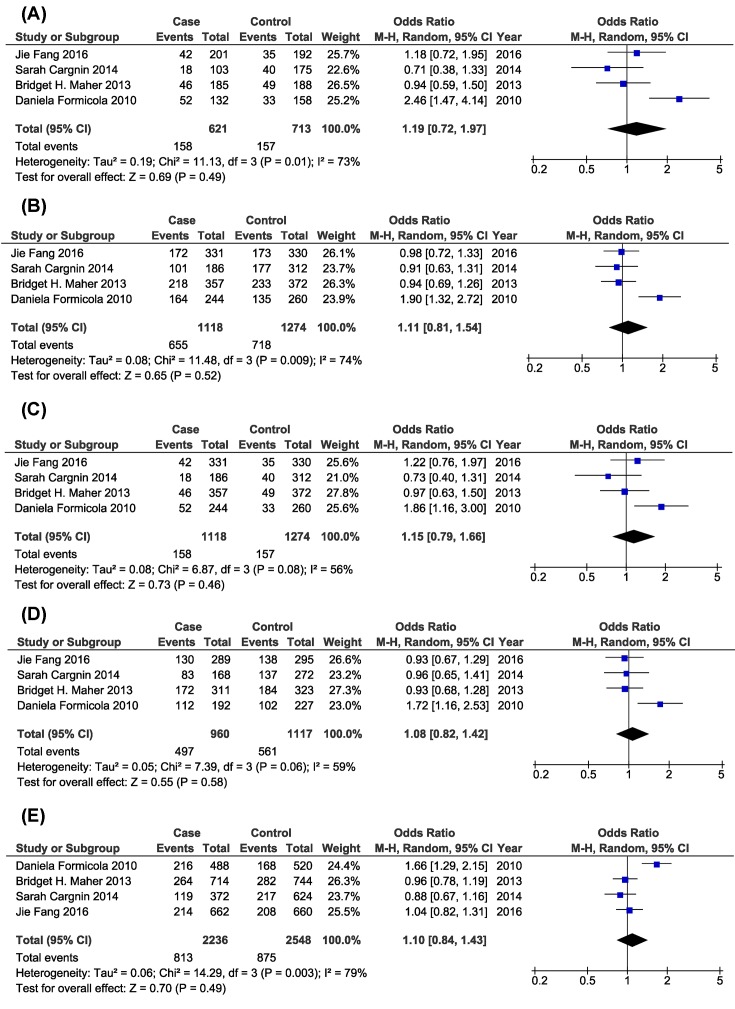
Forest plots of the association of *GRIA1* rs548294 G>A polymorphism and migraine risk (**A**: Homozygous model; **B**: Dominant model; **C**: Recessive model; **D**: Heterozygous model; **E**: Allelic contrast model)

As presented in Figure 2 and Table 3, there was no significant association between *GRIA1* rs548294 G>A polymorphism and migraine risk under all genetic models. Further stratified analysis based on ethnicity also showed no statistical association between *GRIA1* rs548294 G>A polymorphism and migraine risk under all genetic models.

**Table 3:** Meta-analysis of the association between *GRIA1* rs548294 G>A polymorphism and migraine risk

Comparison	Subgroup	*P*_H_	Model	*P*_Z_	OR (95%CI)
Homozygous model (AA vs. GG)	Overall	0.01	R	0.49	1.19 (0.72–1.97)
	Asian	–	–	0.51	1.18 (0.72–1.95)
	Caucasian	0.004	R	0.63	1.19 (0.58–2.46)
Dominant model [(AA+AG) vs. GG]	Overall	0.009	R	0.52	1.11 (0.81–1.54)
	Asian	–	–	0.91	0.98 (0.72–1.33)
	Caucasian	0.004	R	0.51	1.17 (0.74–1.84)
Recessive model [AA vs. (AG+GG)]	Overall	0.08	R	0.46	1.15 (0.79–1.66)
	Asian	–	–	0.40	1.22 (0.76–1.97)
	Caucasian	0.03	R	0.69	1.12 (0.66–1.89)
Heterozygous model (AG vs. GG)	Overall	0.06	R	0.58	1.08 (0.82–1.42)
	Asian	–	–	0.66	0.93 (0.67–1.29)
	Caucasian	0.04	R	0.48	1.14 (0.79–1.67)
Allelic contrast model (A vs. G)	Overall	0.003	R	0.49	1.10 (0.84–1.43)
	Asian	–	–	0.75	1.04 (0.82–1.31)
	Caucasian	0.0009	R	0.55	1.12 (0.77–1.63)

Abbreviations: F, fixed-effects model; R, random-effects model.

**Figure 3:**
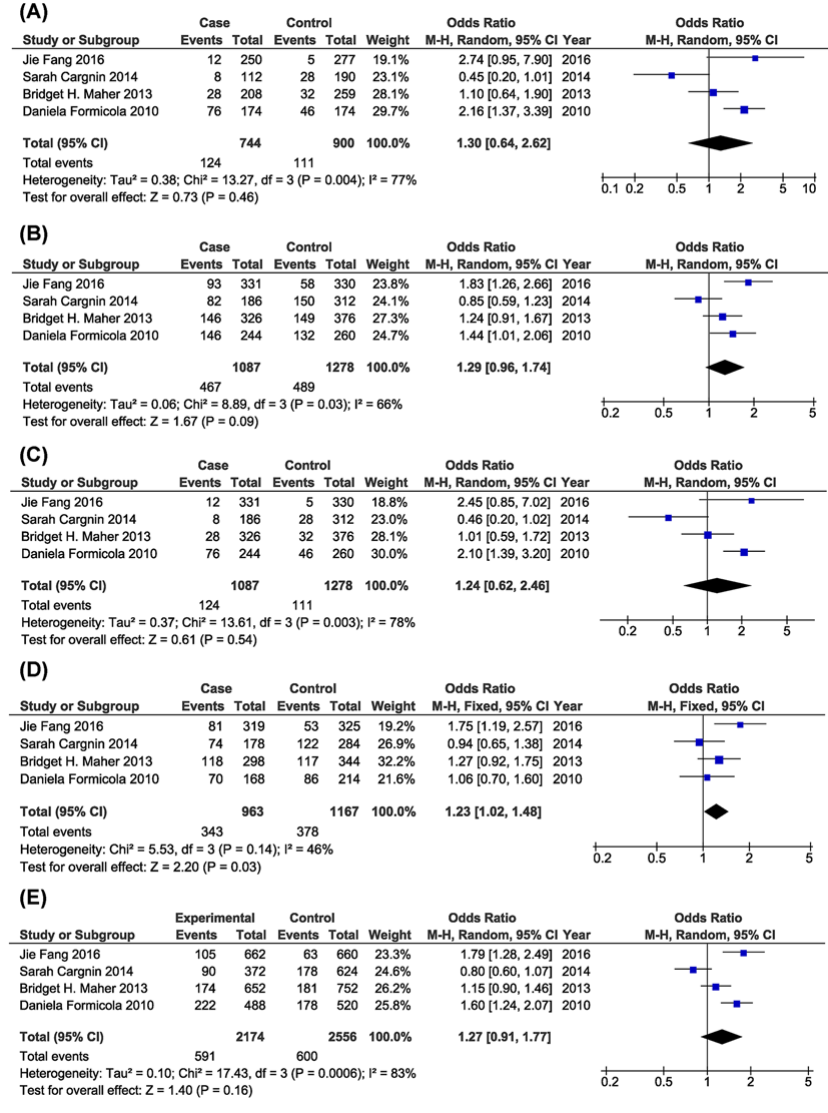
Forest plots of the association of *GRIA1* rs2195450 C>T polymorphism and migraine risk (**A**: Homozygous model; **B**: Dominant model; **C**: Recessive model; **D**: Heterozygous model; **E**: Allelic contrast model)

As shown in Figure 3 and Table 4, there was a significant association between *GRIA1* rs2195450 C>T polymorphism and migraine risk under heterozygous model (CT vs. CC, OR = 1.23, 95%CI = 1.02–1.48, *P*_Z_ = 0.03). In addition, subgroup analysis based on ethnicity showed that *GRIA1* rs2195450 C>T polymorphism was significantly associated with migraine risk in Asian population ((TT+CT) vs. CC, OR = 1.83, 95%CI = 1.26–2.66, *P*_Z_ = 0.001; CT vs. CC, OR = 1.75, 95%CI = 1.19–2.57, *P*_Z_ = 0.005; T vs. C, OR = 1.79, 95%CI = 1.28–2.49, *P*_Z_ = 0.0006), but not in Caucasian population.

**Table 4:** Meta-analysis of the association between *GRIA1* rs2195450 C>T polymorphism and migraine risk

Comparison	Subgroup	*P*_H_	Model	*P*_Z_	OR (95%CI)
Homozygous model (TT vs. CC)	Overall	0.004	R	0.46	1.30 (0.64–2.62)
	Asian	–	–	0.06	2.74 (0.95–7.90)
	Caucasian	0.003	R	0.85	1.08 (0.48–2.46)
Dominant model [(TT+CT) vs. CC]	Overall	0.03	R	0.09	1.29 (0.96–1.74)
	Asian	–	–	0.001	**1.83 (1.26–2.66)**
	Caucasian	0.11	F	0.12	1.17 (0.96**–**1.41)
Recessive model [TT vs. (CT+CC)]	Overall	0.003	R	0.54	1.24 (0.62**–**2.46)
	Asian	–	–	0.10	2.45 (0.85**–**7.02)
	Caucasian	0.002	R	0.91	1.05 (0.47**–**2.37)
Heterozygous model (CT vs. CC)	Overall	0.14	F	0.03	**1.23 (1.02–1.48)**
	Asian	–	–	0.005	**1.75 (1.19–2.57)**
	Caucasian	0.49	F	0.34	1.11 (0.90**–**1.37)
Allelic contrast model (T vs. C)	Overall	0.0006	R	0.16	1.27 (0.91**–**1.77)
	Asian	–	–	0.0006	**1.79 (1.28–2.49)**
	Caucasian	0.002	R	0.48	1.14 (0.79**–**1.67)

Abbreviations: F, fixed-effects model; R, random-effects model. Bold terms indicate significant OR.

### Sensitivity analysis and publication bias

Each study was sequentially removed to assess the impact of single study on the pooled ORs. The result showed that the omission of any one study did not significantly change the overall estimation result on the association between *GRIA1* rs548294 G>A polymorphism and migraine risk. However, after removing Cargnin’s study, the significant association between *GRIA1* rs2195450 C>T polymorphism and migraine risk was observed under homozygous model (TT vs. CC, OR = 1.73, 95%CI = 1.25–2.40, *P*_Z_ = 0.009), heterozygous model (CT vs. CC, OR = 1.33, 95%CI = 1.08–1.65, *P*_Z_ = 0.007), dominant model ((TT+CT) vs. CC, OR = 1.45, 95%CI = 1.19–1.76, *P*_Z_ = 0.0002), and allelic contrast model (T vs. C, OR = 1.46, 95%CI = 1.12–1.91, *P*_Z_ = 0.005). After removing Fang’s study or Formicola’s study, no significant association was observed between *GRIA1* rs2195450 C>T polymorphism and migraine risk under heterozygous model.

Funnel plots were used to assess the publication bias. As shown in Supplementary Figure S1, the funnel plot seemed symmetric and did not show obvious publication bias.

## Discussion

Several genome-wide association studies have identified more than a dozen loci associated with migraine risk, which suggests novel genes and pathways involved in migraine pathophysiology that point toward neuronal and vascular mechanisms of disease [15–17]. In addition, a subsequent genome-wide meta-analysis also revealed five new susceptibility loci for migraine, indicating that considerable increases in the number of cases and controls could further increase the number of risk loci [18].

In 2010, Formicola et al. [7] first reported that *GRIA1* polymorphisms (rs548294 G>A, rs2195450 C>T) were significantly associated with migraine risk in an Italian population. Subsequently, three independent case–control studies from different countries were conducted to confirm this findings [11–13]. The result of their studies argued that *GRIA1* rs548294 G>A polymorphism was not significantly associated with migraine risk. For *GRIA1* rs2195450 C>T polymorphism, the result of Cargnin’s study and Maher’s study was contrary to that of Formicola’s study [7,12,13]. However, the result of Fang’s study indicated that the *GRIA1* rs2195450 C>T polymorphism was significantly associated with migraine risk [11]. These inconsistent results would not be helpful to our understanding of the association between the two polymorphisms and migraine risk. Therefore, an effective method should be adopted to reach a more accurate conclusion.

Meta-analysis is a very powerful tool for analyzing cumulative data of studies, in which the sample sizes are small and the statistical power is poor. In the present study, we used this method to systematically assess the association between *GRIA1* rs548294 G>A and rs2195450 C>T polymorphisms and migraine risk, and found that *GRIA1* rs548294 G>A polymorphism was not significantly associated with migraine risk, but *GRIA1* rs2195450 C>T polymorphism was significantly associated with migraine risk. Due to the fact that rs2195450 polymorphism was located in *GRIA1* intron (NM_000827.3) and had no impact on splicing by the prediction of Human Splicing Finder tool (http://www.umd.be/HSF3/), we inferred that rs2195450 polymorphism was probably not a functional variant. Further linkage analyses for rs2195450 would contribute to finding functional variant associated with migraine risk. Subgroup analysis based on ethnicity showed a significant association of *GRIA1* rs2195450 C>T polymorphism with migraine risk in Asian population, but not in Caucasian population, suggesting that the effect of this polymorphism might depend on genetic background of different ethnicities.

As far as we know, this is the first meta-analysis reporting the association of *GRIA1* rs548294 G>A and rs2195450 C>T polymorphisms and migraine risk. Although the present results could provide stronger evidence for the association, some limitations should not be ignored. First, the number of studies and samples included in the meta-analysis was small and the analysis was based on only four case–control studies, of which three included Caucasian population and one Asian population. The omission of one study, such as Cargnin’s study, Fang’s study or Formicola’s study, could change the overall estimation results of rs2195450 polymorphism under different genetic model, suggesting that the results were not robust and needed to be treated cautiously. In addition, our meta-analysis was based on unadjusted estimates, while a more precise analysis should be performed if individual data such as gender, age, lifestyle and environmental factors were available.

## Conclusion

Our results indicates that *GRIA1* rs2195450 C>T polymorphism is significantly associated with migraine risk. However, more high-quality case–control studies with large sample sizes are still required to confirm these findings.

## Supporting information

**supplementary Figure:** 

## Abbreviations

AMPA: α-amino-3-hydroxy-5-methyl-4-isoxazole propionate
CI: confidence interval
OR: odds ratio
SNP: single-nucleotide polymorphism
