# Supplementary figures and images for: Quantitative assessment of the association between GRIA1 polymorphisms and migraine risk
Source: Biosci Rep. 2018 Dec 14;38(6):BSR20181347. doi: 10.1042/BSR20181347 (PMC6294621; doi:10.1042/BSR20181347)

**rs2195450**

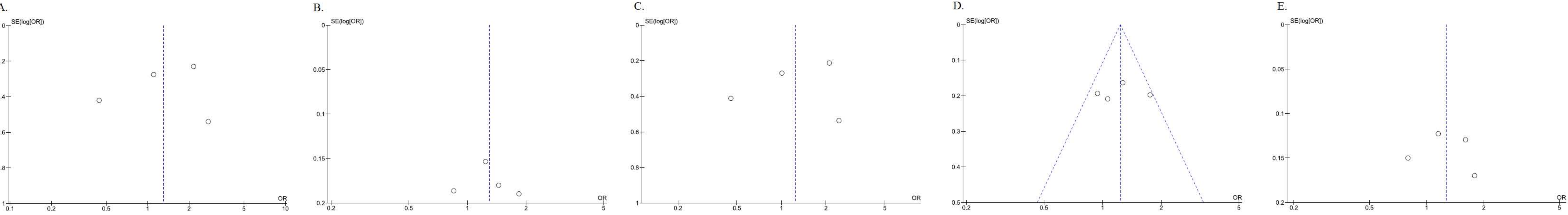

**rs548294**

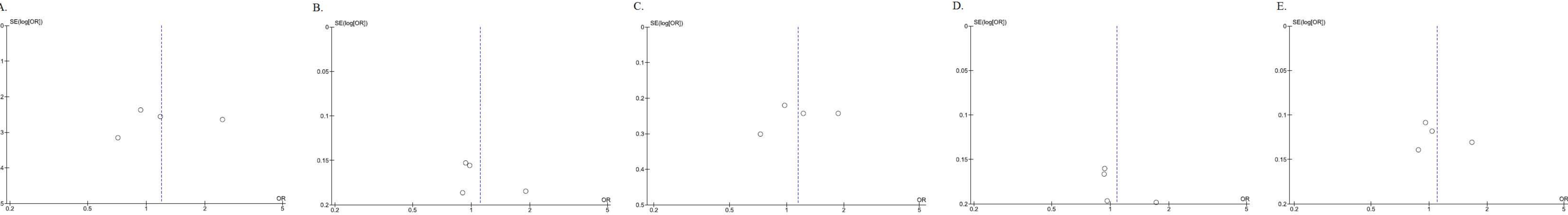

Supplement: Supplementary file 1 [file bsr20181347_Supp1.pdf]
